# Association of kidney fibrosis with urinary peptides: a path towards non-invasive liquid biopsies?

**DOI:** 10.1038/s41598-017-17083-w

**Published:** 2017-12-05

**Authors:** Pedro Magalhães, Martin Pejchinovski, Katerina Markoska, Miroslaw Banasik, Marian Klinger, Dominika Švec-Billá, Ivan Rychlík, Merita Rroji, Arianna Restivo, Giovambattista Capasso, Flaviu Bob, Adalbert Schiller, Alberto Ortiz, Maria Vanessa Perez-Gomez, Pablo Cannata, Maria Dolores Sanchez-Niño, Radomir Naumovic, Voin Brkovic, Momir Polenakovic, William Mullen, Antonia Vlahou, Petra Zürbig, Lars Pape, Franco Ferrario, Colette Denis, Goce Spasovski, Harald Mischak, Joost P. Schanstra

**Affiliations:** 1 Mosaiques Diagnostics GmbH, Hannover, Germany; 2 Department of Pediatric Nephrology, Hannover Medical School, Hannover, Germany; 3 Department of Nephrology, Medical Faculty, University of Skopje, Skopje, Macedonia; 4 Department of Nephrology and Transplantation Medicine, Wroclaw Medical University, Wroclaw, Poland; 5 1st Department of Medicine, Third Faculty of Medicine, Charles University, Prague, Czech Republic; 6 Department of Nephrology, University Hospital Center “Mother Teresa”, Tirana, Albania; 7 Department of Nephrology, University of Campania “Luigi Vanvitelli”, Naples, Italy; 8 Department of Nephrology, ‘Victor Babes’ University of Medicine and Pharmacy, County Emergency Hospital, Timisoara, Romania; 9 IIS-Fundacion Jimenez Diaz UAM, Madrid, Spain; 10 Clinic of Nephrology, Clinical Center of Serbia, Belgrade, Serbia; 11 School of Medicine, University of Belgrade, Belgrade, Serbia; 12 Macedonian Academy of Sciences and Arts, Skopje, Macedonia; 13 Institute of Cardiovascular and Medical Sciences, University of Glasgow, Glasgow, UK; 14 Biotechnology Division, Biomedical Research Foundation, Academy of Athens, Athens, Greece; 15 Nephropathology Center, San Gerardo Hospital, Monza, Italy; 16 Institut National de la Santé et de la Recherche Médicale (INSERM), Institute of Cardiovascular and Metabolic Disease, Toulouse, France; 17 Université Toulouse III Paul-Sabatier, Toulouse, France

## Abstract

Chronic kidney disease (CKD) is a prevalent cause of morbidity and mortality worldwide. A hallmark of CKD progression is renal fibrosis characterized by excessive accumulation of extracellular matrix (ECM) proteins. In this study, we aimed to investigate the correlation of the urinary proteome classifier CKD273 and individual urinary peptides with the degree of fibrosis. In total, 42 kidney biopsies and urine samples were examined. The percentage of fibrosis per total tissue area was assessed in Masson trichrome stained kidney tissues. The urinary proteome was analysed by capillary electrophoresis coupled to mass spectrometry. CKD273 displayed a significant and positive correlation with the degree of fibrosis (Rho = 0.430, *P* = 0.0044), while the routinely used parameters (glomerular filtration rate, urine albumin-to-creatinine ratio and urine protein-to-creatinine ratio) did not (Rho = −0.222; −0.137; −0.070 and *P* = 0.16; 0.39; 0.66, respectively). We identified seven fibrosis-associated peptides displaying a significant and negative correlation with the degree of fibrosis. All peptides were collagen fragments, suggesting that these may be causally related to the observed accumulation of ECM in the kidneys. CKD273 and specific peptides are significantly associated with kidney fibrosis; such an association could not be detected by other biomarkers for CKD. These non-invasive fibrosis-related biomarkers can potentially be implemented in future trials.

## Introduction

Chronic kidney disease (CKD) has become a worldwide problem that affects approximately 10% of the population^1–3^. CKD is defined as chronic anomalies of kidney structure (evidenced by damage markers) leading to a decrease in kidney function and end-stage renal disease (ESRD)^4^. At this stage of CKD, therapeutic interventions are mostly restricted to renal replacement therapy (dialysis or kidney transplantation with life-long immunosuppressive drugs treatments)^5,6^. CKD is also associated with an increased risk to develop cardiovascular complications, and premature death^7^.

Although CKD may develop due to a number of different primary diseases and/or secondary risk factors, progressive tubulo-interstitial fibrosis is one of the most frequent molecular and pathologic features and it is considered a hallmark of CKD^8,9^. Fibrosis is due to the imbalance between extracellular matrix (ECM) synthesis and degradation^8,10^. Renal fibrosis is a significant indicator of disease progression, and affects all kidney structures^11,12^. The pathophysiology of renal fibrosis is associated with an uncontrolled fibrogenesis process, which can be activated by any type of kidney injury. Expansion of fibrosis alters the normal homeostasis and structure of the tissue and results in kidney failure^8,9,13^. At present, there is no effective treatment for CKD. This is in part related to a lack of early markers of kidney disease. Currently, the categorization of CKD is based on the assessment of estimated glomerular filtration rate (eGFR), requiring about 50% of renal function to be lost before a significant reduction can be detected^14^, and/or an increase in the urine-albumin/creatinine-ratio, which can be absent in non-proteinuric causes of CKD^14^. Early detection of fibrotic events may allow earlier detection of CKD and its risk of progression. The current state-of-the-art to assess renal fibrosis is a kidney biopsy which is obviously invasive^15,16^, cannot be repeated frequently, is prone to observer bias and does not enable detailed molecular insight in the molecular components of the deposited ECM^17^.

Focusing on the non-invasive detection of the kidney’s ECM composition could be a good alternative towards the early detection of fibrosis^16^. In this context, the urinary proteome has been extensively studied^18,19^. Particularly, capillary electrophoresis coupled to mass spectrometry (CE-MS), focusing on the low molecular weight urinary proteome, has been successfully used in many studies for the diagnosis and prognosis of CKD^20–25^. An example is CKD273, a multidimensional classifier developed by using CKD-specific peptides present in urine. This classifier is composed of 273 urinary peptides including many peptides (n = 207) derived from the ECM^26^. CKD273 performance has been validated in several independent studies using different cohorts, displaying high sensitivity and specificity for the non-invasive detection of CKD^26–29^. Furthermore, CKD273 enables prediction of progression of CKD^28,30,31^. Indeed, this classifier was able to predict the development of micro- or macroalbuminuria and rapid eGFR loss, demonstrating its utility and advantage over the currently used clinical parameters for predicting CKD progression^30–33^. First data also indicated that CKD273 enables prediction of response to spironolactone^34^. These results have led to the initiation of a randomised controlled clinical trial, PRIORITY, investigating the benefit of CKD273-guided intervention with spironolactone in normoalbuminuric type 2 diabetic patients^35^.

In the current study, we aimed to examine urine and biopsy samples from the same CKD patients, to assess the association of CKD273 with the degree of fibrosis. Furthermore, we also investigated individual urinary peptides potentially correlated with fibrosis.

## Results

### Degree of fibrosis based on Masson trichrome staining

**Figure 1:**
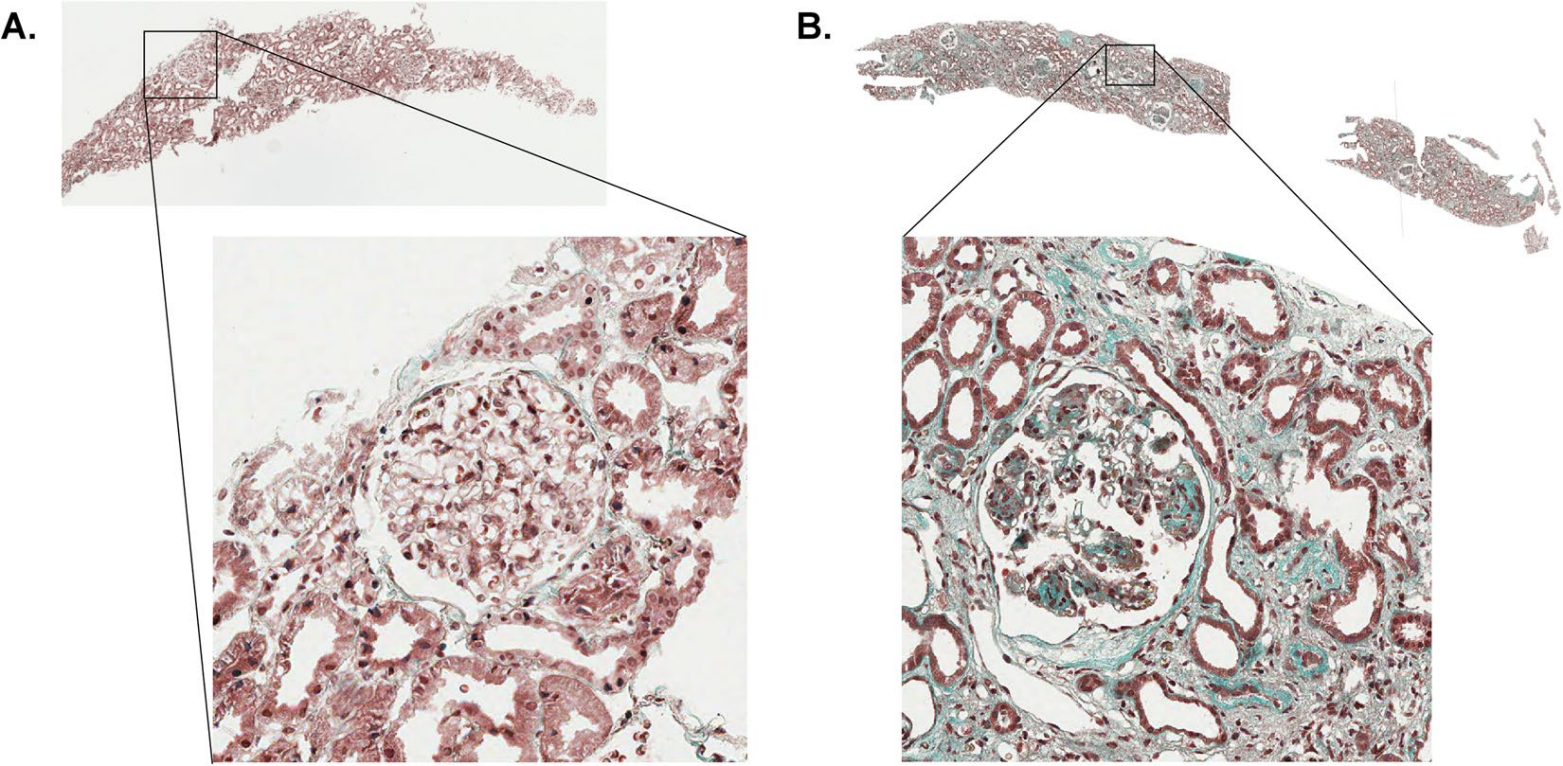
Two examples of biopsies with little (**A**) and massive fibrosis (**B**) as evidenced by Masson trichrome staining (green) that were used for quantification of the degree of fibrosis. (**A**) A patient with mesangiocapillary glomerulonephritis type 2 and (**B**) a patient with mesangiocapillary glomerulonephritis type 1.

Masson trichrome staining was performed on the 42 kidney biopsies to display fibrotic lesions. Two examples are presented in Fig. 1, exemplifying little and massive fibrosis as indicated by the level of green staining. These two examples included patients with mesangiocapillary glomerulonephritis type 2 revealing a reduced fibrosis (Fig. 1A) and with mesangiocapillary glomerulonephritis type 1 showing a massive degree of fibrosis (Fig. 1B).

### Association of CKD273 classifier with the degree of fibrosis

**Figure 2:**
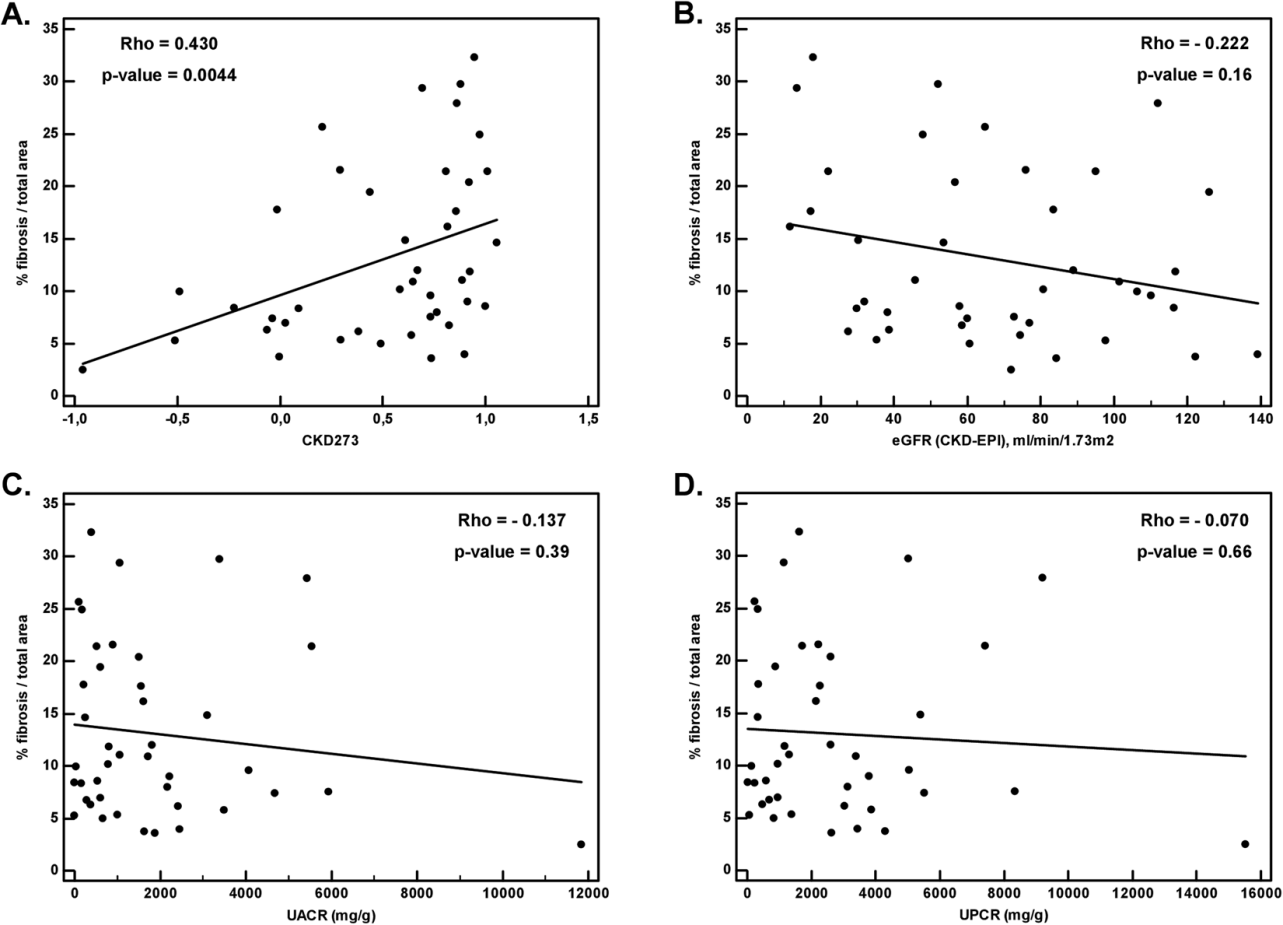
Spearman’s rank correlation analysis, presented by Scatter diagrams. Comparison of different parameters with percentage of fibrosis: (**A**) CKD273 classifier; (**B**) eGFR values (ml/min/1.73 m^2^); (**C**) Urine albumin-to-creatinine ratio (UACR (mg/g)) and (**D**) Urine protein-to-creatinine ratio (UPCR (mg/g)).

We first investigated the association of CKD273 and of the parameters used in routine clinical care to assess the severity of kidney disease (eGFR, urine albumin-to-creatinine ratio and urine protein-to-creatinine ratio values) with the percentage of renal fibrosis per total tissue area of kidney biopsy. As shown in Fig. 2A, CKD273 scores were significantly and positively correlated (Rho = 0.430, *P* = 0.0044) with the degree of fibrosis. A higher CKD273 score had been previously associated with an increased risk of CKD and of CKD progression in larger, non-biopsied cohorts^28,31^. In contrast, none of the other parameters in routine clinical use were significantly correlated to the percentage of fibrosis: eGFR (Rho = −0.222, *P* = 0.16, Fig. 2B), urine albumin-to-creatinine ratio (Rho = −0.137, *P* = 0.39, Fig. 2C) and urine protein-to-creatinine ratio (Rho = −0.070, *P* = 0.66, Fig. 2D).

### Correlation of individual urinary peptides to renal fibrosis

As a second aim, we investigated the correlation of individual urinary peptides with the degree of fibrosis. In total, we assessed 385 urinary peptides that were detected in >50% of all samples and where a high-confidence sequence could be assigned. Out of these 385, seven presented a statistically significant association with the degree of fibrosis (*p* < 0.05) (Table 1). All peptides displayed a negative correlation with fibrosis (−0.552 < Rho <− 0.343) and were ECM related. The peptides were derived from different collagen fragments. Amongst the collagen fragments, six were fibrillar collagens covering type I and III collagens (alpha-1 and 2 chains) and one alpha-1 type XVI collagen, characterized as a fibril-associated forming collagen. Out of these seven urinary peptides, two are also included in the CKD273 classifier and five were previously associated to CKD progression^31^.

**Table 1:** Individual peptides that display a significant correlation with the degree of fibrosis.

Sequence	Protein name	Accession number	StartAA	StopAA	Spearman’s rho	*p-value*	Overlap with CKD273^26^	Previously associated peptides with CKD progression described in Schanstra *et al*.^31^
SpGERGETGPp	Collagen alpha-1(III) chain	P02461	796	806	−0.461	0.007	no	yes
ApGDRGEpGPpGPAG	Collagen alpha-1(I) chain	P02452	798	812	−0.351	0.025	yes	yes
DAGApGApGGKGDAGApGERGpPG	Collagen alpha-1(III) chain	P02461	664	687	−0.343	0.029	no	yes
VGEpGpAGSKGESGNKGEPGSAGP	Collagen alpha-2(I) chain	P08123	345	368	−0.348	0.041	yes	no
ANGAPGNDGAKGDAGAPGApGSQGApG	Collagen alpha-1(I) chain	P02452	699	725	−0.464	0.016	no	yes
PGpPGHPGPpGEPGTDGAAGKEGPpG	Collagen alpha-1(XVI) chain	Q07092	1331	1356	−0.552	0.003	no	no
KNGETGPQGPPGPTGPGGDKGDTGPpGP	Collagen alpha-1(III) chain	P02461	610	637	−0.418	0.043	no	yes

## Discussion

Fibrosis is a hallmark of CKD, however non-invasive specific markers allowing the assessment of the *in situ* ECM content are lacking^16^. Kidney biopsies allow a certain degree of fibrosis assessment but cannot be used to assess the progression of fibrosis in routine clinical care^10,13^. Moreover, fibrosis may be patchy and kidney biopsies may offer an incorrect assessment of the overall degree of fibrosis since tissue is randomly taken and may not be representative of the entire kidney. In fact, this may in part be the reason for the yet significant, but variable association found between CKD273 and the observed fibrosis. However, multiple sampling (*i.e*. repeated biopsies) to improve the accuracy of fibrosis assessment is not possible. Therefore, in this study, we aimed to correlate urinary peptides to the degree of kidney fibrosis with the goal of providing a non-invasive readout of renal fibrosis that may be used to monitor kidney fibrosis.

The most prominent finding of the study was a significant positive correlation of CKD273 with the degree of fibrosis. CKD273 was not initially developed to predict CKD progression and by extension, for the prediction of the degree of fibrosis. However, further studies were conducted^31,36,37^, validating and demonstrating the prognostic value of this classifier. A higher score of CKD273-classifier indicates more severe and advanced CKD. Hence, a positive correlation was expected, due to the fact that CKD273 scores are directly associated with CKD progression^31^. In the case of the seven individual peptides that were correlated to the degree of fibrosis, we hypothesize that the observed negative correlation results from decreased degradation of the kidney ECM proteins, leading to ECM accumulation in the form of kidney fibrosis. Collagen fragments are the most abundant peptides in urine and are thought to be the result of proteolytic activity^8,38^. They are the main structural elements of the interstitial ECM, responsible for cell adhesion, tissue development and tensile strength^39^. The reduced abundance of collagen fragments was previously observed in several studies for different kidney diseases^20,40–42^. Hence, the results presented here are in good agreement with previous findings. Both type I and type III collagens are fibrillary collagens known to form the bulk of kidney fibrosis during kidney scarring. A marked increase in the synthesis of collagen type I, associated with decreased degradation leads to increased collagen deposition in fibrotic glomeruli, tubulointerstitial space and arterial walls^43^. Collagen type III also accumulates in the interstitium and in the glomeruli^44^. A surprising finding was the negative association of type XVI collagen with the degree of fibrosis. Type XVI, a fibril-forming collagen, is expressed in various cells and tissues^45,46^. To our knowledge, this is the first time that this collagen type is associated with CKD progression. However, this potential association is in line with the presence of fibrosis *in-situ*, due to the fact that collagen type XVI is an adaptor protein involved in fibrillar processes, and consequently could contribute to the integrity of the ECM^46,47^.

Enzymes, such as matrix metalloproteinases (MMPs), are responsible for maintaining the homeostasis of the ECM^48^. The underlying molecular process is without doubt rather complex, considering also that not only the activity of the protease, but also the susceptibility of the substrate are relevant in this context. Hence, a situation where post-translational modifications on collagen, e.g. glycation, that effectively inhibit proteolysis by MMPs, could ultimately result in decreased collagen degradation even if MMPs activity was increased^49^.

In hindsight, it is not surprising that routine parameters, used to assess the severity of kidney disease, did not correlate well with kidney biopsy findings. Loss of nephron mass is associated with hyperfiltration, which results in an underestimation of the kidney injury when eGFR is used. However, therapy for kidney disease is aimed to decrease hyperfiltration. The end-result of these factors at the individual patient level is unpredictable and may result in an apparent lack of correlation between eGFR and kidney tissue damage, especially in a study with a low number of subjects, as the one presented here. In addition, mean (SD) single nephron GFR in young humans was 79 ± 42 ml/min and was nearly 40% higher in individuals in their seventies in a study of kidney biopsies from living kidney donors with normal (>90 ml/min) mean GFR^50^. Regarding albuminuria and proteinuria, both proteinuric and non-proteinuric kidney disease share a common pathway of kidney fibrosis. Additionally, anti-proteinuric therapy may further dissociate albuminuria/proteinuria from kidney histology. Furthermore, proximal convoluted tubular uptake of albumin may also be modified in CKD. Up to 30% of diabetic kidney disease cases may develop decreased eGFR without increase of albuminuria^51^.

Fibrosis as a dynamic process is expected to be associated with proteinuria and eGFR^52^. However, in this present study, no significant correlation was found between fibrosis and proteinuria/albuminuria or eGFR. The absence of such correlation may be due to the fact that only one point in time was measured, while fibrosis represents years of progression. The slope of eGFR over time might have displayed a better correlation. In addition, eGFR and albuminuria measurements display a large degree of variability^19^, which could also contribute to the absence of correlation with the degree of fibrosis. Similarly, no association of these clinical parameters with CKD273 was detectable, although described in previous studies^31,53^. This lack of expected associations is thus also likely due to the small sample size. In fact, sample size is a shortcoming of the study. The relatively low patient number is due to the invasive method of obtaining kidney biopsies. Another issue that could be a limitation of this study is the heterogeneity of our cohort, comprising of CKD patients with various etiologies. Different disease processes and risk factors can be associated with a progressive fibrosis. However, this may also be considered as a strength, since a marker that relates to fibrosis independently from the cause of CKD - frequently being unknown- is needed. Another limitation is that we could not obtain the sequence information for all single urinary peptides significantly associated with fibrosis. This can be caused by post-translational modifications of the proteins. Finally, despite kidney biopsy being the current gold standard to assess kidney fibrosis, it involved a very small section (in the range of 0.0025% of total kidney volume) of one of the two kidneys and may not be representative for the full extent of fibrosis, as also indicated above.

In conclusion, our study demonstrated a significant association of CKD273 with the degree of kidney fibrosis, which could not be detected by the current state-of-the-art methods based on serum and urine biochemical parameters used in routine clinical care to estimate the severity of CKD. Furthermore, we identified seven fibrosis-associated peptides that displayed a negative association with the degree of fibrosis. To our knowledge, the present study provides the first investigation of CKD273 and urinary peptides related to the severity of kidney fibrosis. Additional studies in larger cohorts are required to further validate these potential associations. However, the results of the present study are in line with previous observations suggesting that CKD273 is a good predictor of CKD progression.

## Subjects and Methods

### Patient Cohort

Matched urine and kidney tissue samples were collected from seven different centers: Wroclaw Medical University (Wroclaw, Poland; n = 13), Clinical Center of Serbia (Belgrade, Serbia; n = 12), Charles University (Prague, Czech Republic; n = 7), IIS-Foundation Jimenez Diaz (Madrid, Spain; n = 4), University Hospital Center “Mother Teresa” (Tirana, Albania; n = 3), University of Campania “Luigi Vanvitelli” (Naples, Italy; n = 2), and University of Medicine and Pharmacy Timisoara (Timisoara, Romania; n = 1). The patient cohort consisted of 42 CKD patients. Patients’ clinical data (eGFR, albuminuria and proteinuria) were measured based on the urine samples, which were collected on the same day as the kidney biopsies were performed. Albuminuria (mg/g) was determined by the ratio of the measured urine albumin and urine creatinine. Proteinuria was calculated in a similar way, however, based on urine-protein values. Baseline eGFR (mL/min/1.73 m^2^) was estimated using the CKD-EPI formula. CKD stages were defined on the basis of the eGFR, allowing the following distribution of the patients: 26.2% in category G1, eGFR ≥90 ml/min/1.73 m^2^; 23.8% in G2, eGFR 60–89 ml/min/1.73 m^2^; 35.7% in G3, eGFR 30–59 ml/min/1.73 m^2^; 11.9% in G4, eGFR 15–29 ml/min and 2.4% in G5, eGFR <15 ml/min/1.73 m^2^, according to the KDIGO categorization system^4^. This cohort was constituted by patients of different CKD etiologies: chronic hypertensive nephropathy (n = 1); Henoch-Schönlein Purpura - Nephritis (HSPN; n = 1); Idiopathic Rapid Progressive Glomerulonephritis (n = 1); IgA Nephropathy (IgAN; n = 9); Membranous Glomerulonephritis (MGN; n = 1); Mesangioproliferative Glomerulonephritis (n = 4); Mesangiocapillary Glomerulonephritis (n = 7); Minimal Change disease (MCD; n = 3); Primary Focal Segmental Glomerulosclerosis (FSGS; n = 3), Tubulointerstitial Nephritis (TIN, n = 1); Membranous Nephropathy (MN; n = 5), Minimal Change Nephropathy (n = 4); Systemic vasculitis-ANCA positive (n = 1) and Transplant Glomerulopathy (n = 1).

Baseline characteristics of the patients included in this study are summarized in Table 2.

**Table 2:** Baseline characteristics of the study population.

Characteristics	
Number of subjects	42
Age (years)	43.99 ± 17.72
Gender (F/M)	19/23
**Mean ± SD of characteristic**	
Systolic BP (mmHg)	131.62 ± 17.20
Dyastolic BP (mmHg)	80.64 ± 10.03
Mean BP (mmHg)	97.63 ± 10.79
UACR (mg/g)	1882.49 ± 2250.21
UPCR (mg/g)	2773.68 ± 3045.47
eGFR (CKD-EPI). mL/min/1.73 m²	67.28 ± 34.55
CKD273 scores	0.51 ± 0.48
% fibrosis/total area	13.07 ± 8.25

BP: blood pressure; UACR: urine albumin-to-creatinine ratio; UPCR: urine protein-to-creatinine ratio; eGFR: estimated glomerular filtration rate

The study was designed and conducted in accordance with the standards of good clinical practice and principles of the Helsinki Declaration. Written informed consent was obtained from all participants. The protocol was approved by each center local ethics committee (Ethics Committee of the Hospital of the Second University of Naples, Italy; Multicentric ethics committee of the Faculty Hospital Kralovske Vinohrady, Czech Republic; Component of the Local Ethics Commission for Scientific Research of Timisoara, County Emergency Clinical Hospital, Romania; National Ethics Committee from the Ministry of Health, Republic of Albania; Bioethics Committee at the Medical University of Wroclaw, Poland; Ethics Committee of Clinical Center of Serbia, Serbia; Ethics Committee of Clinical Center of the IIS-Foundation Jimenez Diaz, Spain) and the general one from the coordinating center in Skopje, Macedonia - Ethic Subcommittee for Medicine, Pharmacy, Veterinary and Stomatology-Macedonian Academy of Science and Arts (Ethical ID: 09-1785/5).

### Urine samples

#### Sample preparation and CE-MS analysis

Urine sample collection and CE-MS analysis were carried out as previously described^26,54^. Briefly, 0.7 ml of urine was thawed and diluted with 0.7 ml of a solution containing 2 M urea, 0.1 M NaCl, 10 mM NH4OH and 0.02% SDS. The sample was filtered using a Centrisart ultracentrifugation filter devices (20 kDa molecular weight cut-off; Sartorius, Goettingen, Germany) at 2,600xg for one hour at 4 °C until 1.1 ml filtrate was obtained. Subsequently, the filtrate was applied onto a PD-10 desalting column (GE Healthcare, Sweden) equilibrated in 0.01% aqueous NH4OH. Finally, the eluate was lyophilized, stored at 4 °C prior to be resuspended in HPLC-grade water for CE-MS analysis.

CE-MS analysis was performed using a P/ACE MDQ capillary electrophoresis system (Beckman Coulter, Fullerton, USA) online coupled to a MicroTOF MS (BrukerDaltonic, Bremen, Germany)^55^. The ESI sprayer (Agilent Technologies, Palo Alto, CA, USA) was grounded, and the ion spray interface potential was set between −4 and −4.5 kV. Spectra were accumulated every 3 seconds over a range of mass-to-charge from 50 to 3000. Details on accuracy, precision, selectivity, sensitivity, reproducibility and stability of the CE-MS method were previously reported in detail^55^.

#### Data processing

MosaiquesVisu was used to deconvolute mass spectral peaks representing identical molecules into singles masses. The obtained peak list of each polypeptide is characterized by molecular mass [in Daltons], CE-migration time (in minutes), and normalized ion signal intensity. MS signal intensities were used as a measure of relative abundance and normalized using 29 internal standard peptides (peptides generally present in at least 90% of all urine)^56^. All detected peptides were deposited, matched and annotated in a Microsoft SQL database^57^, permitting further correlation and statistical analysis.

#### Peptide Sequencing

To obtain the sequence information, tandem mass spectrometry was performed using a Dionex Ultimate 3000 RSLC nano flow system (Dionex, Camberly, UK) or a Beckman CE systems (PACE MDQ) coupled to an Orbitrap Velos MS instrument (Thermo Fisher Scientific)^58,59^. Data files were analyzed using Proteome Discoverer 1.2 and were searched against the UniProt human non-redundant database without enzyme specificity and with hydroxylation of proline and lysine, as well as oxidation of methionine. The assessment of the sequences was based on the molecular mass and calculated CE-migration time based on its peptide sequence (number of basic amino acids). Peptides were accepted only if they had a mass deviation below ±5 ppm and <50 mDa for the fragment ions. In addition, CE-migration time deviation was required to be below ±2 min.

#### Tissue samples and Image analysis

Sections from Formalin Fixed Paraffin Embedded (FFPE) blocks with bioptic tissues were shipped to Nephropathology Department of San Gerardo Hospital in Monza where histopathological diagnoses and scores were performed according to the predefined renal scoring system for glomerular, vascular and interstitial changes^60^.

Biopsy slides stained with Masson trichrome were scanned with an Aperio Scanner (Leica Biosystems) and analyzed with ImageJ software (version 1.51n, https://imagej.nih.gov/ij/). The surface occupied by the green marker was evaluated as fibrosis surface. Based on these measurements, the level of fibrosis, expressed as a percentage of the total measured area, was calculated. The degree of fibrosis was calculated according to the following equation: % fibrosis per total area = (fibrosis area/total tissue area) * 100. Total area is defined by the total kidney tissue area that is available to evaluate for image analysis.

#### Correlation and statistical analysis

The non-parametric Spearman’s rank correlation coefficient was used to estimate the correlation of the CKD273 classifier with the degree of fibrosis. The same method was applied to investigate the correlation between the parameters routinely used in the clinic to assess the severity of kidney damage and to categorize CKD based on the risk of CKD progression and premature mortality (eGFR, urine albumin-to-creatinine ratio, urine protein-to-creatinine ratio)^4^ and the percentage of fibrosis. Correlations were considered significant when displaying a *P value* <0.05. These analyses were performed using MedCalc software (version 12.1.0.0; MedCalc Software, Mariakerke, Belgium).

To determine the correlation of individual peptides with the percentage of fibrosis as a continuous variable, Spearman’s rank correlation was assessed, because the peptide profiles across the samples are not normally distributed. Peptides present in at least 50% (frequency threshold) of the samples were included in the correlation analysis. The analysis was performed using R-based statistic software and confirmed by MedCalc. Graphs were generated with MedCalc.

### Data availability

The datasets generated during and/or analysed during the current study are available from the corresponding author on reasonable request.
